# Sensorless Impedance Control of Micro Finger Using Coprime Factorization

**DOI:** 10.3390/mi16050510

**Published:** 2025-04-27

**Authors:** Yuuki Morohoshi, Mingcong Deng

**Affiliations:** Department of Electrical Engineering and Computer Science, Graduate School of Engineering, Tokyo University of Agriculture and Technology, 2-24-16 Nakacho, Koganei-shi 184-8588, Tokyo, Japan; ymorohoshi.res@gmail.com

**Keywords:** coprime factorization, force control, impedance control, nonlinear control, micro finger, observer, operator theory, robust control, soft actuator, Youla–Kucera parametrization

## Abstract

Soft robots are attracting attention as next-generation robots because they enable flexible movement. The micro finger is a soft robot that can bend and is small and can grasp objects of various shapes, so it is expected to be applied to surgical robots. However, because it is small, sensors cannot be attached, making it difficult to measure force. This paper proposes impedance control of the tip of a micro finger by estimating the tip force with an observer. The control system is designed using coprime factorization and Youla–Kucera parameterization by operator theory. The effectiveness of the proposed method is confirmed through experiments.

## 1. Introduction

Recently, Japan has become one of the world’s leading aging societies with a declining birthrate, and the decrease in the working population is a major concern [1]. The medical and nursing care fields are most affected by the labor shortage, and this issue is expected to become even more severe in the future. Therefore, the demand for robots in the medical and welfare fields is increasing. Traditional robots have been developed for predetermined simple tasks such as assembling and sorting manufactured goods, as well as for exploring dangerous areas such as planetary exploration and high-radiation environments. However, robots in the medical and welfare fields are required to perform precise movements, flexible movements, and ensure safety for tasks such as surgery, internal examinations, and carrying people. These requirements are difficult to achieve with conventional actuators such as motors and cylinders alone, leading to increased attention on soft actuators. Soft actuators are actuators that can operate under physical perturbations due to their physical shape and flexible materials [2]. Examples of soft actuators include pneumatic actuators [3,4] and shape memory alloy actuators [5,6]. Recently, soft actuators of various shapes have been proposed using 3D printers, and many robots using soft actuators have also been studied [7,8].

The micro finger used in this study is a pneumatic soft actuator with a hollow semi-cylindrical shape, consisting of a bellow structure on the arc side and a flat surface on the other side [4]. It can bend by generating torque through the increase and decrease of air pressure. Due to its simple structure, small size, and flat shape, it is expected to be used in micro robots and surgical robots, and research in this area is actively being conducted [4,9,10,11]. Wakimoto et al. [4] developed a silicone rubber micro finger and analyzed its displacement and force characteristics. Furthermore, they connected three micro fingers to create a robot hand capable of grasping objects. Zhao et al. [9] proposed a micro finger using McKibben-type artificial muscles and confirmed that it generates approximately 8.5 times the force compared to a silicone micro finger. Polygerinos et al. [10] created various shapes of micro fingers, proposed models, and analyzed their displacement characteristics, providing a series of systematic design rules useful for creating micro fingers. Despite various studies, the presence of nonlinear elements such as rubber and pneumatic pressure makes position and force control challenging due to significant variations in response to loads.

Therefore, many researchers have studied this topic [1,12,13,14,15,16]. Most studies use PID control, but due to the strong nonlinearity of the micro finger, it is difficult to address safety concerns [12,13,14]. Consequently, efforts have been made to safely control the micro finger using coprime factorization [1,15,16]. Deng et al. [15] created a model of the micro finger using machine learning and performed sensorless control using coprime factorization. However, it is necessary to create a new model each time the environment changes because there is no way to grasp the position when the actual device is operating. Bu et al. [16] modeled the hysteresis characteristics of the rubber and compressed air of the micro finger using the Generalized Prandtl–Ishlinskii model (GPI model), designed a control system using coprime factorization based on isomorphism and terminal sliding mode control (TSMC), and confirmed its effectiveness through simulations. Isomorphism requires feedback of the quasi-state using sensors to simplify the mathematical model, but when sensors are not available, it is necessary to use an observer for feedback, which can degrade control performance if there are uncertainties in the mathematical model. Therefore, the authors in [1] designed a control system using coprime factorization for complex mathematical models without using isomorphism and confirmed its effectiveness through experiments. As mentioned above, sufficient research has been conducted on the position control of the micro finger. While precise position control of the micro finger is important, it is even more crucial to grasp objects with appropriate force. Although the force generated at the tip of the micro finger has been analyzed, sufficient research has not been conducted on force control. This is likely due to the lack of sensors that can be attached to the small tip of the micro finger.

In this study, we propose sensorless impedance control of the micro finger using coprime factorization. Since it is difficult to attach sensors directly to the micro finger, we estimate the external force from the tip position and applied air pressure. By using coprime factorization, we ensure system stability and achieve safe control. The effectiveness of the proposed method is confirmed through actual device experiments.

The structure of this paper is as follows. Section 2 shows the micro finger and impedance control. Next, Section 3 presents the problem setting, specifically the issues with the micro finger and the research direction. Section 4 designs the control system, followed by Section 5, which presents the results of the actual device experiments. Finally, Section 6 concludes and discusses future prospects.

## 2. Preliminaries

This section explains the details of the micro finger and provides an overview of impedance control. For an explanation of coprime factorization and Youla–Kucera parameterization, please refer to [17,18,19].

### 2.1. Micro Finger

#### 2.1.1. Overview of Micro Finger

**Figure 1:**
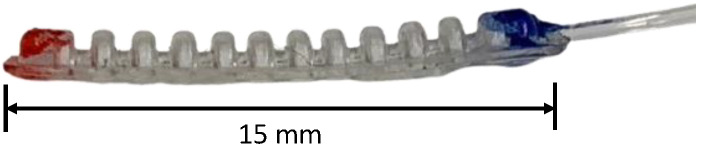
Micro finger.

The micro finger used in this study is shown in Figure 1. It is a pneumatic actuator made by combining a bellow structure and a flat structure of silicone rubber [4]. It curves inwards on its flat surfaces when pressure is increased, and curves inwards on its bellows surfaces when subjected to a vacuum.

**Figure 2:**
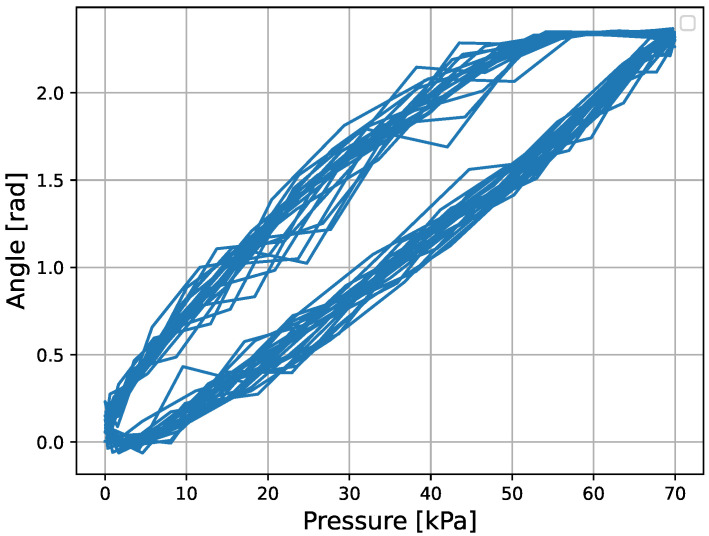
Hysteresis characteristics of the micro finger.

The micro finger is composed of rubber and compressed air, which exhibit hysteresis characteristics. Figure 2 shows the change in the bending angle of the micro finger when a sinusoidal wave with an amplitude of 35 kPa, a frequency of 0.4 Hz, and a bias of 35 kPa is applied.

**Figure 3:**
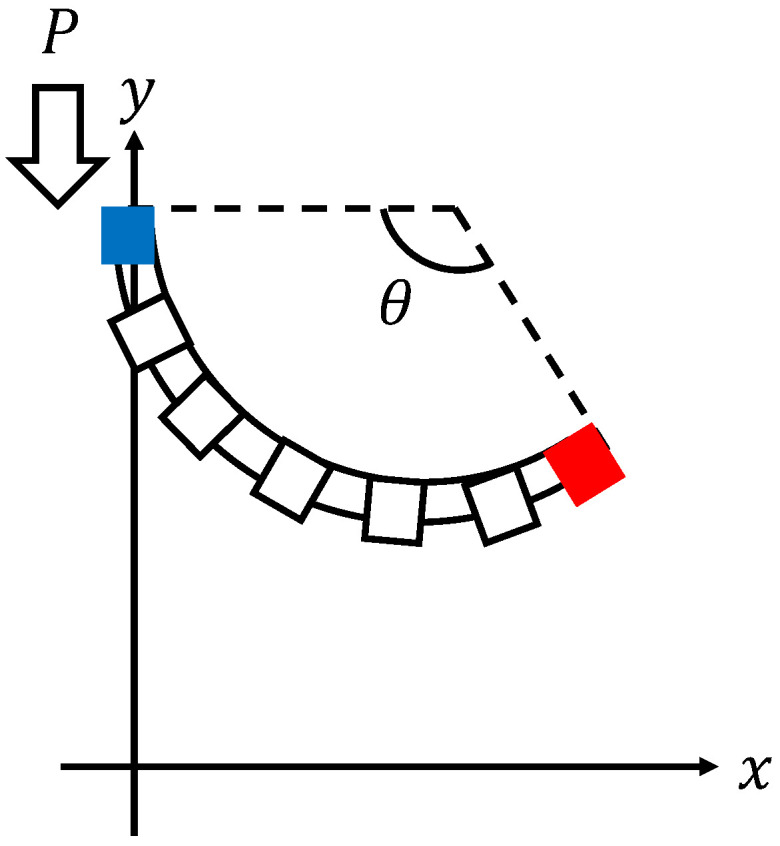
Coordinate system of the micro finger.

The coordinate system of the micro finger is set as shown in Figure 3.

In this study, the output y(t) is the bending angle θ(t), and the control input u(t) is the pressure P(t). From previous studies [20], the mathematical model of the micro finger is expressed as:

(1) $$\begin{array}{c} P\left(u\right)\left(t\right):\left\{\begin{array}{c} \dot{x}\left(t\right)=\alpha \left(-x(t)-\gamma +\beta u(t)\right)\\ y\left(t\right)=\frac{n}{2C_{1}}\left(C_{2}-\sqrt{C_{2}^{2}-4C_{1}C_{3}h_{PI}\left(x\right)\left(t\right)}\right)+C_{4} \end{array}\right., \end{array}$$

where the state variable *x* is the pressure inside the micro finger. Also, $C_{1},C_{2},C_{3}$ are expressed as:

(2) $$\begin{array}{cc} \; & C_{1}=\frac{R_{2}^{4}-R_{1}^{4}}{2l^{2}}, \end{array}$$

(3) $$\begin{array}{cc} \; & C_{2}=\frac{k_{C_{2}}\pi \left(R_{2}^{3}-R_{1}^{3}\right)}{4l}, \end{array}$$

(4) $$\begin{array}{cc} \; & C_{3}=\frac{4\left(r_{2}^{3}-{\left(r_{1}+t_{th}\right)}^{3}\right)}{Et_{th}}, \end{array}$$

where the natural length $L_{0}$ of the micro finger is assumed to be constant. The parameters are shown in Table 1. Also, $C_{4}>0$ is the bending angle when the input is 0 kPa. Additionally, $h_{PI}\left(x\right)\left(t\right)$ is a function representing hysteresis characteristics, using the concept of the GPI model as in previous study [16,21], treating the hysteresis characteristics as linear and residual terms:

(5) $$\begin{array}{c} h_{PI}\left(x\right)\left(t\right)=k_{PI}x\left(t\right)+\Delta_{PI}\left(t\right), \end{array}$$

where $k_{PI}$ is the proportional gain. In this study, to simplify the control system design, we assume $\Delta_{PI}\left(t\right)=0$. To simplify the notation of the plant, f,g,h are defined as:

(6) $$\begin{array}{cc} \; & f\left(x\right)\left(t\right)=-\alpha \left(x\left(t\right)+\gamma \right), \end{array}$$

(7) $$\begin{array}{cc} \; & g=\alpha \beta , \end{array}$$

(8) $$\begin{array}{cc} \; & h\left(x\right)\left(t\right)=\frac{n}{2C_{1}}\left(C_{2}-\sqrt{C_{2}^{2}-4C_{1}C_{3}h_{PI}\left(x\right)\left(t\right)}\right)+C_{4}. \end{array}$$

In this case, Equation (1) can be expressed as:

(9) $$\begin{array}{c} P\left(u\right)\left(t\right):\left\{\begin{array}{c} \dot{x}\left(t\right)=f\left(x\right)\left(t\right)+gu\left(t\right)\\ y\left(t\right)=h\left(x\right)\left(t\right) \end{array}\right.. \end{array}$$

In addition, from a previous study [22], the external force $f_{e}\left(t\right)$ applied to the tip of the micro finger is expressed as:

(10) $$\begin{array}{c} f_{e}\left(t\right)=\frac{M_{r}\left(t\right)-M_{p}\left(t\right)}{\frac{L_{0}}{y^{2}\left(t\right)}\left(1-\cos y(t)\right)}, \end{array}$$

where $M_{r}\left(t\right)$ and $M_{p}\left(t\right)$ are the moments due to elastic force and pressure, respectively. Note that y(t)≠0. Their difference is expressed as:

(11) $$\begin{array}{c} M_{r}\left(t\right)-M_{p}\left(t\right)=Et_{th}\left(C_{1}{\left(\frac{y\left(t\right)-C_{4}}{n}\right)}^{2}-C_{2}\frac{y\left(t\right)-C_{4}}{n}+C_{3}h_{PI}\left(x\right)\left(t\right)\right). \end{array}$$

#### 2.1.2. Experimental Equipment

**Figure 4:**
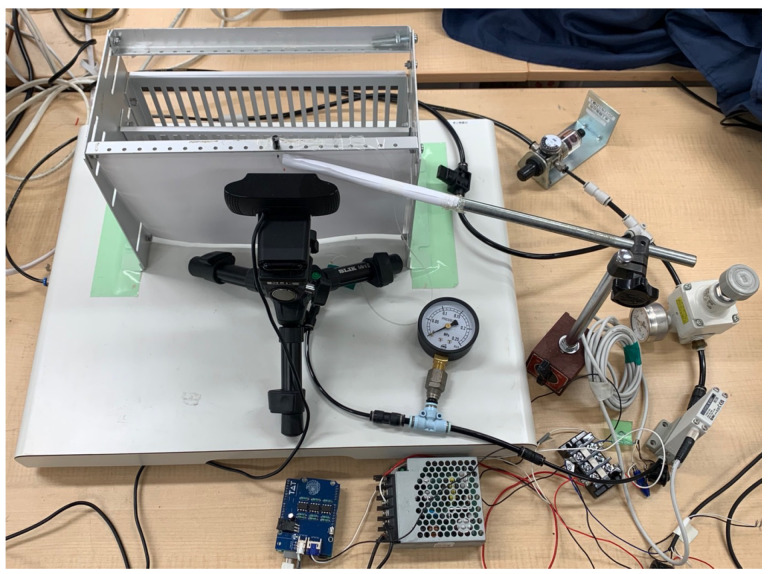
Experimental equipment for the micro finger.

**Figure 5:**
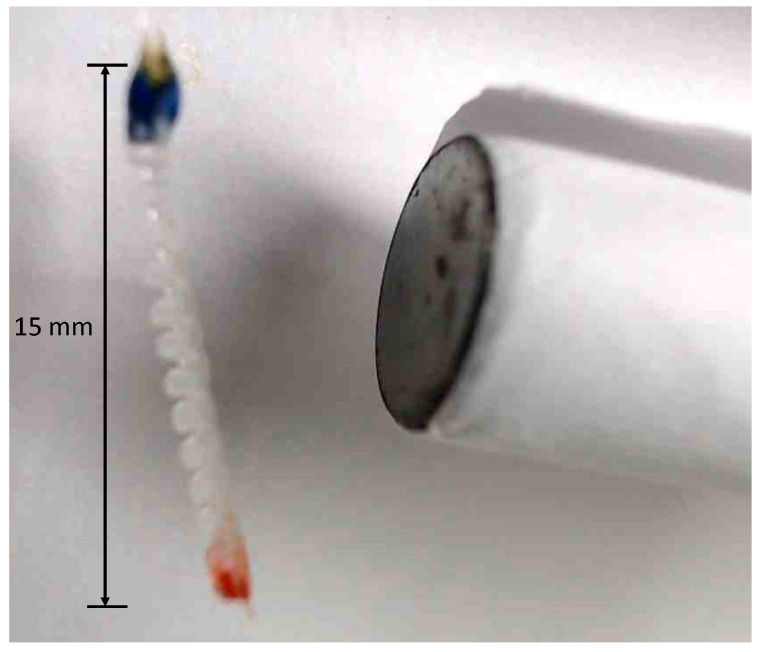
Enlarged view of Figure 4.

The experimental equipment for the micro finger is shown in Figure 4 and Figure 5. Compressed air generated from the compressor is filtered to remove dust, moisture, and oil by the filter regulator, then the pressure is limited to below 100 kPa by the safety regulator, and finally sent to the electro-pneumatic regulator. The electro-pneumatic regulator sends compressed air to the micro finger according to the command value from the PC. Impedance control is performed by placing a rod so that the micro finger makes contact when it bends, as shown in Figure 5. Also, the micro finger is placed on a vibration isolation table to suppress vibrations from the compressor and other sources.

The measurement of the tip coordinates of the micro finger is performed by a camera. The camera reads the red tip coordinates $\left(x_{t}\left(t\right),y_{t}\left(t\right)\right)$ shown in Figure 3, and the angle y(t) is calculated using the following equation:

(12) $$\begin{array}{c} y\left(t\right)=2\cos^{-1}\frac{L_{0}-y_{t}\left(t\right)}{\sqrt{x_{t}{\left(t\right)}^{2}+{\left(L_{0}-y_{t}\left(t\right)\right)}^{2}}}. \end{array}$$

### 2.2. Impedance Control

**Figure 6:**
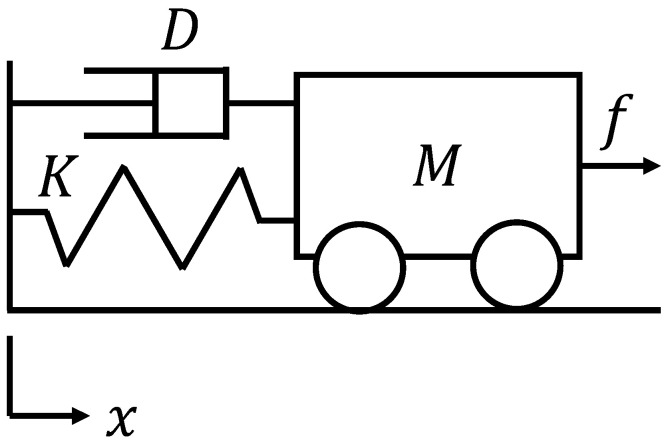
Overview of mechanical impedance.

Impedance control is a control method that virtually controls the weight of an object [23]. As shown in Figure 6, an object has three elements: mass M≥0, viscosity D≥0, and elasticity K≥0. The relationship between the position x(t) of the object and the force f(t) applied to the object is determined by these elements as shown in Equation (13). Furthermore, this relationship can be generalized using an operator as shown in:

(13) $$\begin{array}{cc} \; & f\left(t\right)=M\ddot{x}\left(t\right)+D\dot{x}\left(t\right)+K\left(x\left(t\right)-x_{0}\right), \end{array}$$

(14) $$\begin{array}{cc} \; & f\left(t\right)=Z\left(x\left(t\right)-x_{0}\right), \end{array}$$

where $x_{0}$ is the position where f(t)=0 when the object is at rest. The parameters M,D,K, and Z(·) represent the relationship between the position and force of the object, similar to how impedance in an electrical circuit represents the relationship between current and voltage. Therefore, this is called mechanical impedance.

Next, consider the case where an actuator such as a motor is attached to the object. In this case, the force f(t) applied to the object is expressed as the sum of the force $f_{m}\left(t\right)$ by the actuator and the external force $f_{e}\left(t\right)$, as shown in:

(15) $$\begin{array}{c} f\left(t\right)=f_{m}\left(t\right)+f_{e}\left(t\right), \end{array}$$

where, since $f_{m}\left(t\right)$ is the force by the actuator, its value can be freely determined by the program. Therefore, the value of $f_{m}\left(t\right)$ is expressed using virtual mass $\hat{M}\geq 0$, elasticity $\hat{K}\geq 0$, and viscosity $\hat{D}\geq 0$, as shown in:

(16) $$\begin{array}{c} f_{m}\left(t\right)=\left(M-\hat{M}\right)\ddot{x}\left(t\right)+\left(D-\hat{D}\right)\dot{x}\left(t\right)+\left(K-\hat{K}\right)\left(x\left(t\right)-x_{0}\right). \end{array}$$

Then, using Equations (13) and (15), the relationship between the position x(t) of the object and the external force $f_{e}\left(t\right)$ is expressed as shown in:

(17) $$\begin{array}{c} f_{e}\left(t\right)=\hat{M}\ddot{x}\left(t\right)+\hat{D}\dot{x}\left(t\right)+\hat{K}\left(x\left(t\right)-x_{0}\right). \end{array}$$

This allows the impedance of the object to be virtually changed from M,D,K to $\hat{M},\hat{D},\hat{K}$. This control method is applied to devices that assist the human body, such as powered suits and electric bicycles, due to the stability and ease of imposing limits on the force $f_{m}\left(t\right)$ by the actuator and the feature of controlling the “weight” of the object.

## 3. Problem Setting

When the micro finger grasps an object, it is necessary to ensure that it does not apply excessive force and damage the object. However, since it is difficult to attach a force sensor to the tip of the micro finger, sufficient research on force control of the micro finger has not been conducted. Therefore, this study proposes an impedance control system for the micro finger using coprime factorization. In this study, we estimate and control the external force transmitted from the micro finger to the object. The external force estimation is applied to the micro finger using Equation (10). However, since the external force estimator also considers the uncertainty of hysteresis, a hysteresis model is incorporated into the controller design to prevent this. Additionally, the hysteresis characteristics of the micro finger are assumed to be due to pneumatic pressure and are divided into linear and residual terms based on the concept of the GPI model. Furthermore, for the simplification of the control system design, we assume $\Delta_{PI}\left(t\right)=0$ in this study.

## 4. Control System Design

**Figure 7:**
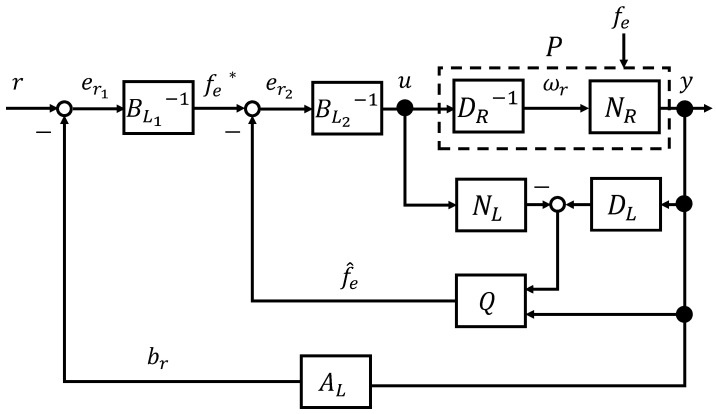
Impedance control system of the micro finger.

The control system for the impedance control of the micro finger is shown in Figure 7. Since it is difficult to attach a force sensor to the micro finger itself, the position read by the camera is input to the external force estimator, and the estimated external force ${\hat{f}}_{e}\left(t\right)$ is fed back.

### 4.1. Right Coprime Factorization

Hereafter, the control system design is carried out assuming $f_{e}\left(t\right)=0$. The stable operator $A_{L}$ must feedback the position, so it is designed as shown in:

(18) $$\begin{array}{c} A_{L}\left(b\right)\left(t\right):b\left(t\right)=y\left(t\right). \end{array}$$

Since the operator $B_{L_{1}}^{-1}$ is an impedance controller, the stable and invertible operator $B_{L_{1}}$ is represented as shown in:

(19) $$\begin{array}{c} B_{L_{1}}\left({f_{e}}^{*}\right)\left(t\right):\left\{\begin{array}{c} {\ddot{x}}_{b_{1}}\left(t\right)=-k_{1}{\dot{x}}_{b_{1}}\left(t\right)-k_{2}x_{b_{1}}\left(t\right)+k_{3}e_{r_{1}}\left(t\right)\\ e_{r_{1}}\left(t\right)=\frac{1}{K}\left(-M{\ddot{x}}_{b_{1}}\left(t\right)-D{\dot{x}}_{b_{1}}\left(t\right)+{f_{e}}^{*}\left(t\right)\right) \end{array}\right., \end{array}$$

where $k_{1},k_{2},k_{3}>0$ are design parameters. Also, the operator $B_{L_{2}}$ is designed as shown in:

(20) $$\begin{array}{c} B_{L_{2}}\left(u\right)\left(t\right):\left\{\begin{array}{c} {\dot{x}}_{b_{3}}\left(t\right)=f\left(x_{b_{3}}\right)\left(t\right)+gu\left(t\right)\\ y_{b}\left(t\right)=h\left(x_{b_{3}}\right)\left(t\right)\\ x_{b_{2}}\left(t\right)=h\left(\beta u\left(t\right)-\gamma \right)-y_{b}\left(t\right)\\ e_{r_{2}}\left(t\right)=Kx_{b_{2}}\left(t\right) \end{array}\right.. \end{array}$$

When $e_{r_{2}}\left(t\right)={f_{e}}^{*}\left(t\right)$, the operator $B_{L}$ is represented as shown in:

(21) $$\begin{array}{c} B_{L}\left(u\right)\left(t\right):\left\{\begin{array}{c} {\dot{x}}_{b_{3}}\left(t\right)=f\left(x_{b_{3}}\right)\left(t\right)+gu\left(t\right)\\ y_{b}\left(t\right)=h\left(x_{b_{3}}\right)\left(t\right)\\ x_{b_{2}}\left(t\right)=h\left(\beta u\left(t\right)-\gamma \right)-y_{b}\left(t\right)\\ e_{r_{2}}\left(t\right)=Kx_{b_{2}}\left(t\right)\\ {f_{e}}^{*}\left(t\right)=e_{r_{2}}\left(t\right)\\ {\ddot{x}}_{b_{1}}\left(t\right)=-k_{1}{\dot{x}}_{b_{1}}\left(t\right)-k_{2}x_{b_{1}}\left(t\right)+k_{3}e_{r_{1}}\left(t\right)\\ e_{r_{1}}\left(t\right)=\frac{1}{K}\left(-M{\ddot{x}}_{b_{1}}\left(t\right)-D{\dot{x}}_{b_{1}}\left(t\right)+{f_{e}}^{*}\left(t\right)\right) \end{array}\right.. \end{array}$$

Therefore, the stable operator $N_{R}$ and the stable and invertible operators $D_{R}$ are designed as shown in:

(22) $$\begin{array}{c} N_{R}\left(\omega_{r}\right)\left(t\right):\left\{\begin{array}{c} {\ddot{x}}_{b_{1}}\left(t\right)=-k_{1}{\dot{x}}_{b_{1}}\left(t\right)-k_{2}x_{b_{1}}\left(t\right)+k_{3}\left(\omega_{r}\left(t\right)-y\left(t\right)\right)\\ {f_{e}}^{*}\left(t\right)=M{\ddot{x}}_{b_{1}}\left(t\right)+D{\dot{x}}_{b_{1}}\left(t\right)+K\left(\omega_{r}\left(t\right)-y\left(t\right)\right)\\ e_{r_{2}}\left(t\right)={f_{e}}^{*}\left(t\right)\\ x_{b_{2}}\left(t\right)=\frac{1}{K}e_{r_{2}}\left(t\right)\\ {\dot{x}}_{b_{3}}\left(t\right)=f\left(x_{b_{3}}\right)\left(t\right)+gu\left(t\right)\\ y_{b}\left(t\right)=h\left(x_{b_{3}}\right)\left(t\right)\\ u\left(t\right)=\frac{1}{\beta }\left(h^{-1}\left(x_{b_{2}}+y_{b}\right)\left(t\right)+\gamma \right)\\ \dot{x}\left(t\right)=f\left(x\right)\left(t\right)+gu\left(t\right)\\ y\left(t\right)=h\left(x\right)\left(t\right) \end{array}\right., \end{array}$$

(23) $$\begin{array}{cc} \; & D_{R}\left(\omega_{r}\right)\left(t\right):\left\{\begin{array}{c} {\ddot{x}}_{b_{1}}\left(t\right)=-k_{1}{\dot{x}}_{b_{1}}\left(t\right)-k_{2}x_{b_{1}}\left(t\right)+k_{3}\left(\omega_{r}\left(t\right)-y\left(t\right)\right)\\ {f_{e}}^{*}\left(t\right)=M{\ddot{x}}_{b_{1}}\left(t\right)+D{\dot{x}}_{b_{1}}\left(t\right)+K\left(\omega_{r}\left(t\right)-y\left(t\right)\right)\\ e_{r_{2}}\left(t\right)={f_{e}}^{*}\left(t\right)\\ x_{b_{2}}\left(t\right)=\frac{1}{K}e_{r_{2}}\left(t\right)\\ {\dot{x}}_{b_{3}}\left(t\right)=f\left(x_{b_{3}}\right)\left(t\right)+gu\left(t\right)\\ y_{b}\left(t\right)=h\left(x_{b_{3}}\right)\left(t\right)\\ u\left(t\right)=\frac{1}{\beta }\left(h^{-1}\left(x_{b_{2}}+y_{b}\right)\left(t\right)+\gamma \right) \end{array}\right.. \end{array}$$

From Equations (22) and (23), the operators $N_{R}$ and $D_{R}$ are stable. Then, the stability of the system is guaranteed. When $e_{r_{2}}\left(t\right)={f_{e}}^{*}\left(t\right)$, Equation (24) is obtained for the closed loop of Figure 7:

(24) $$\begin{array}{cc} A_{L}N_{R}\left(\omega_{r}\right)\left(t\right)+B_{L_{1}}B_{L_{2}}D_{R}\left(\omega_{r}\right)\left(t\right) & =h\left(x\right)\left(t\right)+\left(\omega_{r}-h\left(x\right)\right)\left(t\right),\\ \; & =\omega_{r}\left(t\right). \end{array}$$

Therefore, since $M_{R}\left(\omega_{r}\right)\left(t\right)=\omega_{r}\left(t\right)$, the system is stable.

### 4.2. External Force Estimator

The design of the external force estimator is carried out. The stable operator $N_{L}$ and the stable and invertible operator $D_{L}$ are designed as shown in:

(25) $$\begin{array}{c} N_{L}\left(u\right)\left(t\right):\left\{\begin{array}{c} {\dot{x}}_{n_{l}}\left(t\right)=f\left(x_{n_{l}}\right)\left(t\right)+gu\left(t\right)\\ b_{l}\left(t\right)=-Et_{th}C_{3}\left(x_{n_{l}}\right)\left(t\right) \end{array}\right., \end{array}$$

(26) $$\begin{array}{cc} \; & D_{L}\left(y\right)\left(t\right):e_{l}\left(t\right)=Et_{th}\left(C_{1}{\left(\frac{y-C_{4}}{n}\right)}^{2}-C_{2}\frac{y-C_{4}}{n}\right). \end{array}$$

The operators $N_{L}$ and $D_{L}$ are left coprime factorizations, and it is confirmed that the moment of the external force can be extracted from (11). Assuming $\hat{d}\left(t\right)$ is the moment of the external force, the operator *Q* is designed as shown in Equation (27) from Equation (10).

(27) $$\begin{array}{c} Q\left(\begin{array}{c} \hat{d}\\ y \end{array}\right)\left(t\right):{\hat{f}}_{e}\left(t\right)=\frac{\hat{d}\left(t\right)}{\frac{L_{0}}{y{\left(t\right)}^{2}}\left(1-\cos y(t)\right)}, \end{array}$$

Note that the following holds:

(28) $$\begin{array}{c} Q\left(\begin{array}{c} 0\\ y \end{array}\right)\left(t\right):{\hat{f}}_{e}\left(t\right)=\frac{0}{\frac{L_{0}}{y^{2}\left(t\right)}\left(1-\cos y(t)\right)}=0. \end{array}$$

### 4.3. System Stability with External Force Estimator

The stability of the system is guaranteed when the external force estimator is added to Equation (24). The estimated external force ${\hat{f}}_{e}\left(t\right)$ is expressed as:

(29) $$\begin{array}{cc} {\hat{f}}_{e}\left(t\right) & =Q\left(\begin{array}{c} \hat{d}\\ y \end{array}\right)\left(t\right). \end{array}$$

When $f_{e}\left(t\right)=0$, $\hat{d}\left(t\right)$ is expressed as:

(30) $$\begin{array}{cc} \hat{d}\left(t\right) & =D_{L}\left(y\right)\left(t\right)-N_{L}\left(u\right)\left(t\right),\\ \; & =D_{L}N_{R}\left(\omega_{r}\right)\left(t\right)-N_{L}D_{R}\left(\omega_{r}\right)\left(t\right),\\ \; & =0. \end{array}$$

Therefore, from Equation (27), ${\hat{f}}_{e}\left(t\right)=0$. Thus, the system is stable even when the external force estimator is added to Equation (24) by Youla–Kucera parameterization.

### 4.4. Proof of Tracking Performance

When the force reference input $f_{e}^{*}\left(t\right)$ is applied such that ${\dot{f}}_{e}^{*}{\left(t\right)|}_{t\to \infty }=0$, it is shown that $e_{r_{2}}\left(t\right){|}_{t\to \infty }=0$ when a sufficient amount of time has passed. When a sufficient amount of time has passed and the derivatives of all state variables are 0, the operator $B_{L_{2}}$ is represented as shown in:

(31) $$\begin{array}{c} B_{L_{2}}{\left(u\right)\left(t\right)|}_{t\to \infty }:\left\{\begin{array}{c} x_{b_{3}}\left(t\right)=\beta u\left(t\right)-\gamma \\ y_{b}\left(t\right)=h\left(x_{b_{3}}\right)\left(t\right)\\ x_{b_{2}}\left(t\right)=h\left(\beta u\left(t\right)-\gamma \right)-y_{b}\left(t\right)\\ e_{r_{2}}\left(t\right)=Kx_{b_{2}}\left(t\right) \end{array}\right.. \end{array}$$

In Equation (31), $e_{r_{2}}$(t) is represented as shown in:

(32) $$\begin{array}{cc} e_{r_{2}}{\left(t\right)|}_{t\to \infty } & =Kx_{b_{2}}{\left(t\right)|}_{t\to \infty },\\ \; & =K\left(h\left(\beta u\left(t\right)-\gamma \right)-y_{b}\left(t\right)\right){|}_{t\to \infty },\\ \; & =K\left(h\left(\beta u\left(t\right)-\gamma \right)-h\left(x_{b_{3}}\right)\left(t\right)\right){|}_{t\to \infty },\\ \; & =K\left(h\left(\beta u\left(t\right)-\gamma \right)-h\left(\beta u\left(t\right)-\gamma \right)\right){|}_{t\to \infty },\\ \; & =0. \end{array}$$

Therefore, when the force reference input $f_{e}^{*}\left(t\right)$ is applied such that ${\dot{f}}_{e}^{*}{\left(t\right)|}_{t\to \infty }=0$, it is shown that $e_{r_{2}}{\left(t\right)|}_{t\to \infty }=0$ when a sufficient amount of time has passed.

## 5. Experimental Results

**Figure 8:**
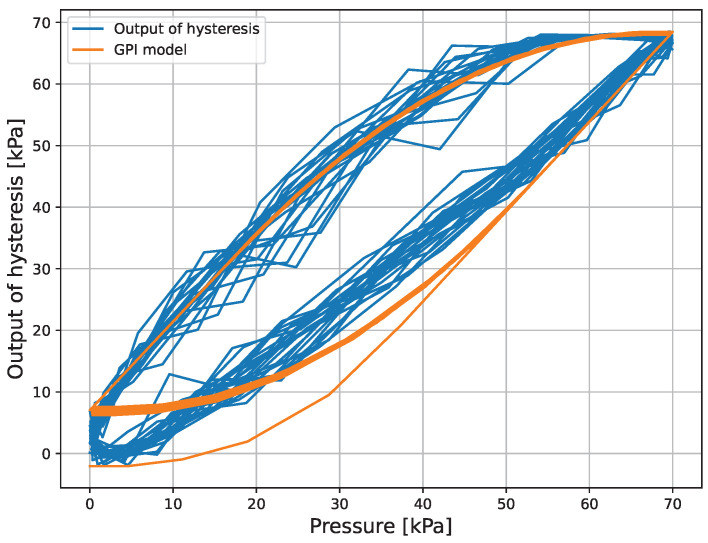
Fitting result.

The results of the force control experiment of the micro finger are shown. The parameters used in the experimental experiment are shown in Table 2. Here, the gain $k_{PI}$ of the GPI model was obtained by converting the data in Figure 2 to pressure using the inverse model of Equation (8) and applying the least squares method. The results of the fitting are shown in Figure 8.

**Figure 9:**
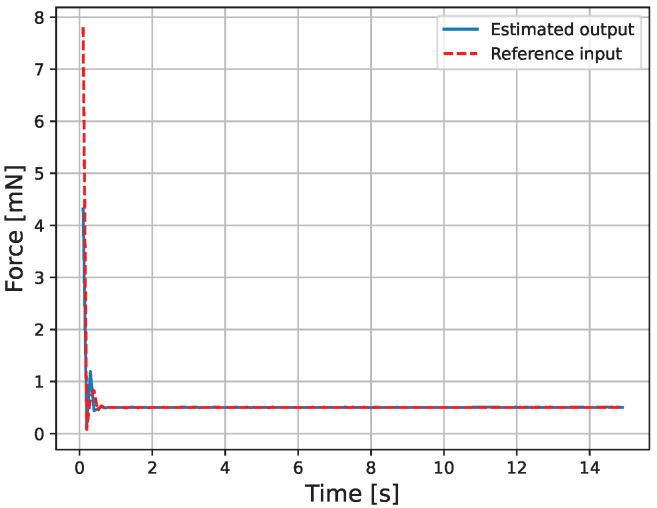
Force.

**Figure 10:**
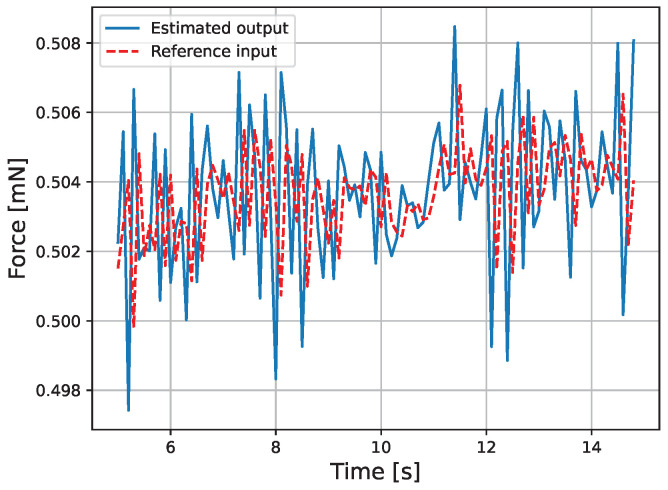
Enlarged view of the force.

**Figure 11:**
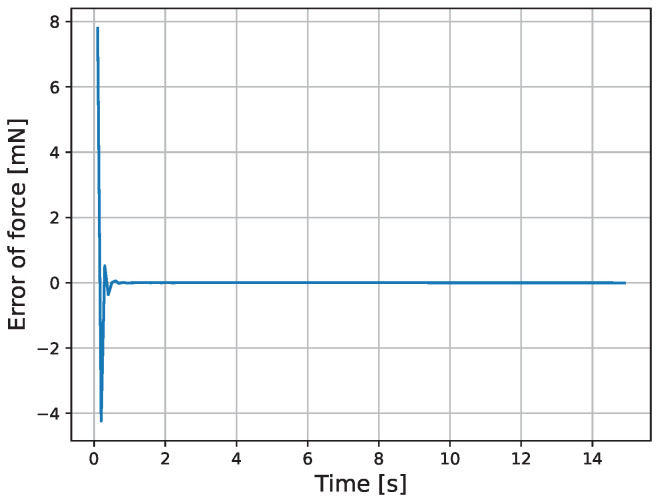
Force error.

**Figure 12:**
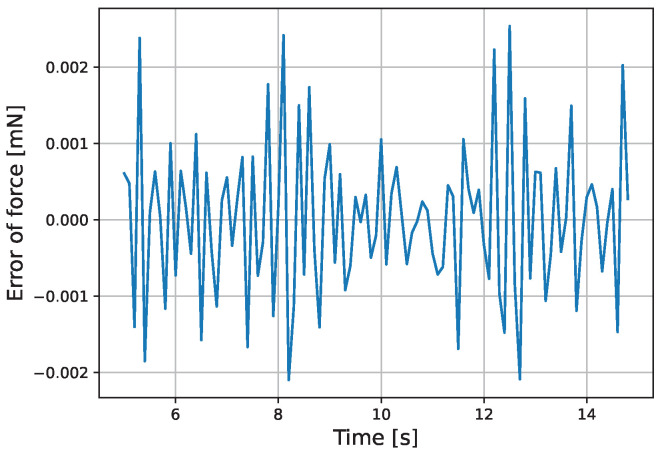
Enlarged view of the force error.

**Figure 13:**
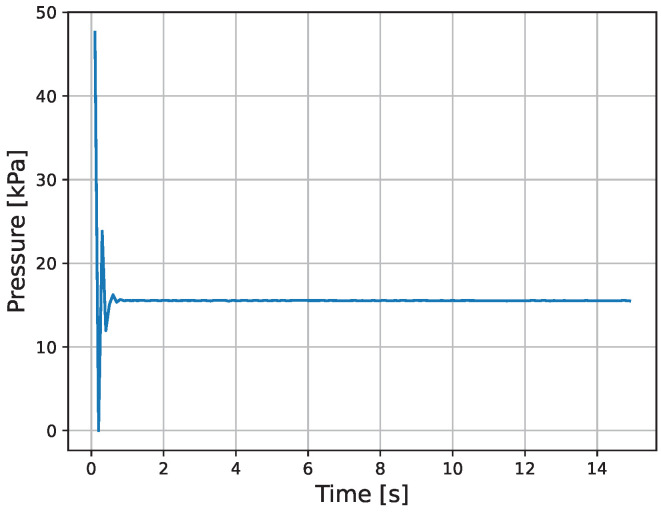
Pressure.

**Figure 14:**
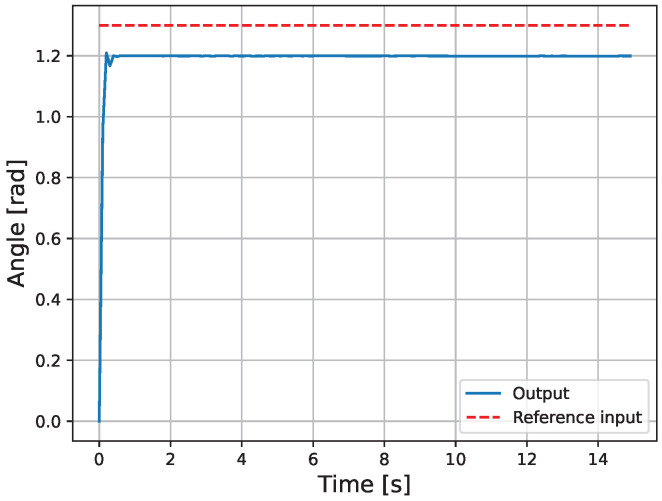
Bending angle.

The results of the actual experiment are shown in Figure 9, Figure 10, Figure 11, Figure 12, Figure 13 and Figure 14. Note that Figure 10 and Figure 12 are enlarged views of Figure 9 and Figure 11, respectively. From Figure 9 and Figure 10, it can be confirmed that the estimated external force of the micro finger follows the reference input, although there is a slight delay. This result is considered reasonable because the input to the micro finger does not saturate, as shown in Figure 13, and the bending angle does not follow as shown in Figure 14.

### Discussion

We discuss the reason why the estimated external force ${\hat{f}}_{e}^{*}\left(t\right)$ responds faster than the force reference value $f_{e}^{*}\left(t\right)$ in Figure 10. The force reference value $f_{e}^{*}\left(t\right)$ is the output of the operator $B_{L_{1}}$, and from Equation (19), the output y(t) is affected by the delay of the second-order low-pass filter in the operator $B_{L_{1}}$. Therefore, the force reference value $f_{e}^{*}\left(t\right)$ experiences a delay in its response. On the other hand, the estimated external force ${\hat{f}}_{e}^{*}\left(t\right)$ is the output of the operator *Q*, and from Equation (27), the output y(t) is not affected by the delay of a low-pass filter in the operator *Q*. As a result, the estimated external force ${\hat{f}}_{e}^{*}\left(t\right)$ responds faster than the force reference value $f_{e}^{*}\left(t\right)$.

We discuss the oscillation of the force reference value $f_{e}^{*}\left(t\right)$ observed in Figure 10. This is considered to be due to measurement deviations caused by camera noise. Since the operator $B_{L_{1}}$, which outputs the force reference value $f_{e}^{*}\left(t\right)$, uses a second-order low-pass filter, as shown in Equation (19), the influence of oscillations due to the differentiation of ${\ddot{x}}_{b_{1}}\left(t\right)$ and ${\dot{x}}_{b_{1}}\left(t\right)$ is considered to be small. Therefore, it is thought that the oscillation is caused by the term of the position deviation $e_{r_{1}}\left(t\right)$, and since the position reference value r(t) is a step signal, the output y(t) is oscillating. Hence, it is speculated that the noise is due to the exposure amount and other factors during camera measurement. To solve this problem, it is necessary to introduce an observer to remove the noise. However, since the properties of the micro finger are susceptible to changes in the environment and may also remove external forces, an adaptive observer needs to be considered.

## 6. Conclusions

This paper proposed sensorless impedance control of a micro finger using coprime factorization. Since the micro finger is very small and it is difficult to attach sensors, the external force was estimated using an observer from the tip position measured by a camera. In the actual experiment, the force reference input showed a delay compared to the estimated external force of the micro finger, and the force reference input oscillated. The former is considered to be due to phase delay caused by the presence of the low-pass filter. The latter is considered to be caused by noise from the camera, so it is necessary to remove the noise using an observer. In the future, we will consider a compensator that can solve the above two points simultaneously.

## Figures and Tables

**Table 1:** Parameters for the micro finger.

Parameter	Description	Unit
*E*	Young’s modulus	[Pa]
$L_{0}$	Natural length	[m]
*l*	Initial length of the one bellows	[m]
*n*	Number of the bellows	[–]
$R_{1}$	Representative radius of the small chambers	[m]
$R_{2}$	Representative radius of the large chambers	[m]
$r_{1}$	Initial radius of the small chambers	[m]
$r_{2}$	Initial radius of the large chambers	[m]
$t_{th}$	Thickness of the rubber	[m]
α	Parameter of the control valve	[-]
β	Parameter of the control valve	[-]
γ	Parameter of the control valve	[Pa]
$k_{C_{2}}$	Correction factor	[-]

**Table 2:** Parameters for the experimental experiment.

Symbol	Description	Value	Unit
$C_{4}$	Angle when input is 0 kPa	1.2	rad
*E*	Young’s modulus	$0.95\times 10^{6}$	Pa
$L_{0}$	Natural length	$15\times 10^{-3}$	m
*l*	Natural length of the one bellows	$0.6\times 10^{-3}$	m
*n*	Number of bellows	12	–
$R_{1}$	Representative radius of the small chambers	$0.325\times 10^{-3}$	m
$r_{1}$	Initial radius of the small chambers	$0.25\times 10^{-3}$	m
$R_{2}$	Representative radius of the large chambers	$0.925\times 10^{-3}$	m
$r_{2}$	Initial radius of the large chambers	$0.85\times 10^{-3}$	m
$t_{th}$	Thickness of the rubber	$0.15\times 10^{-3}$	m
α	Parameter of the control valve	6	1/s
β	Parameter of the control valve	0.3	–
γ	Parameter of the control valve	$3.1\times 10^{3}$	Pa
$k_{C_{2}}$	Correction factor	5.61	–
$k_{PI}$	Gain of hysteresis characteristics	0.96	–
$T_{s}$	Sampling time	0.1	s
$\bar{u}$	Maximum control input	$7.0\times 10^{4}$	Pa
$\underline{u}$	Minimum control input	0	Pa
*D*	Virtual damping coefficient	$5\times 10^{-5}$	N·s/m
*K*	Virtual stiffness	$5\times 10^{-3}$	N/m
*M*	Virtual mass	$5\times 10^{-6}$	g
$k_{1}$	Design parameter of $B_{L_{1}}$	2	1/s
$k_{2}$	Design parameter of $B_{L_{1}}$	1	1/s^2^
$k_{3}$	Design parameter of $B_{L_{1}}$	1	1/s^2^

## Data Availability

The data presented in this study are available in this article.
